# Epitopes for Protective Immunity Targeting Antigens of Pathogen and/or Host (EPITAPH): Towards Novel Vaccines Against HIV and Other Medically Challenging Infections

**DOI:** 10.3389/fimmu.2014.00270

**Published:** 2014-06-10

**Authors:** Salvador Eugenio C. Caoili

**Affiliations:** ^1^Department of Biochemistry and Molecular Biology, College of Medicine, University of the Philippines Manila, Manila, Philippines

**Keywords:** epitopes, immunity, antibodies, pathogens, hosts, vaccines, HIV, AIDS

Antiretroviral therapy (ART) currently enables long-term survival of persons living with HIV (PLWH), but the respite from escalating pandemic AIDS-related deaths is undermined by both emergence of drug-resistant HIV and failure to develop HIV vaccines (1). Such reliance on drugs instead of vaccines is arguably a defeatist strategy against infectious diseases in the face of inevitable pathogen drug resistance. Still, the development of anti-infective vaccines is presently constrained by the exclusion of host self epitopes from candidate vaccine components. This aims to avoid inducing autoreactive host immune responses, some of which might yet be exploited to prevent or control infection by disrupting key host–pathogen interactions, notably using antibodies that bind critical epitopes and thereby sterically hinder molecular recognition (e.g., between virus and host cell). Classic vaccine-induced antibody responses are intended to target only pathogen-derived antigens or analogs thereof (e.g., recombinant or synthetic fragments); however, pathogen immune evasion may occur especially where epitope variability generates immunodominant decoy epitopes that elicit non-protective and possibly even harmful (e.g., infection-enhancing) antibody responses. Although vaccine design might be attempted to elicit protective antibody responses only against carefully selected pathogen epitopes, this is biologically unrealistic if based on a reductionist approach whereby individual epitopes are evaluated in isolation from one another, neglecting their functional interdependence in the context of host infection and immunity (2). Extending the notion of synergistic simultaneous targeting of structurally distinct epitopes, both pathogen-derived and host self epitopes are plausible targets of antibody-mediated protective immunity. As both pathogen and host contribute to pathogenesis of infectious disease (3), vaccination potentially can limit overall host damage due to both pathogen-associated (i.e., virulence) and host-associated (i.e., immune) factors, with some degree of host-induced damage being acceptable in place of more extensive pathogen-induced damage. An HIV vaccine thus might elicit antibodies that bind the gp120 receptor (CD4) or co-receptors (CCR4 and CCR5) to interrupt the viral replication cycle. This would be self-defeating if it resulted in excessive harm due to autoimmune host damage (e.g., manifest as quantitative or qualitative deficits of CD4+ cells, resulting in severe immunodeficiency), but tantalizing alternative scenarios are suggested by cases of natural resistance to HIV-1 infection that feature anti-CD4 autoantibodies (4).

Although binding of host self antigens by antibodies risks host damage, such binding may occur without resulting in appreciable harm. In support of this view, an apparent lack of pathological manifestations has been noted among healthy individuals who developed circulating antiplatelet autoantibodies (e.g., binding the platelet glycoprotein complex gpIb-IX) subsequent to immunization with recombinant HIV gp160 (5); and certain rare broadly neutralizing HIV-1 antibodies (e.g., to the 2F5 and 4E10 epitopes of HIV gp41) have been shown to exhibit polyspecific autoreactivity [e.g., such that the 2F5 and 4E10 antibodies bind the host phospholipid cardiolipin (6) yet also bind other human autoantigens including kynureninase and splicing factor 3b subunit 3, respectively (7)]. Such binding of host self antigens by autoantibodies entails broken self tolerance, which is typically difficult to induce (consistent with the rarity of 2F5- and 4E10-like antibodies, presumably reflecting an evolutionary adaptation that avoids autoimmune host damage); but in spite of this, immunization with recombinant constructs comprising CCR5-derived sequences recently has been shown (using murine and simian models) to elicit apparently non-deleterious anti-CCR5 antibody responses that block HIV (8) or SIV (9) infectivity, which points to the prospect of developing safe and efficacious HIV vaccines that induce protective immunity based on antibody targeting of judiciously selected host self epitopes (rather than whole autoantigens).

Hence, functional epitope mapping conceivably could delineate host self epitopes as targets for antibody binding *in vivo*, to block infection without producing excessive damage. Such targeting of host self epitopes might be sufficient to block infection (e.g., if binding of the epitopes by antibodies in itself imposed steric hindrance that directly precluded crucial biomolecular interactions); otherwise, infection still might be blocked by simultaneous binding of both host self- and pathogen-associated epitopes by synergistically acting antibodies (e.g., with sufficient steric hindrance realized only between host- and pathogen-bound antibodies). Furthermore, host self epitopes tend to be highly conserved, which could facilitate vaccine design for entire host populations. The risk–benefit trade-off posed by host-reactive antibody-mediated immunity could be explored initially through passive immunization with monoclonal antibodies (mAbs), before committing to active-immunization strategies (e.g., that employ prophylactic or therapeutic vaccines). The mAbs could be developed in tandem with specific antidotes (e.g., anti-idiotypic constructs that prevent antibody binding of host self epitopes) for treating any adverse reactions that might occur, for instance, due to effector mechanisms such as complement activation and antibody-dependent cell-mediated cytotoxicity (ADCC). Antibody-mediated complement activation might be minimized by avoiding the juxtaposition of target epitopes (e.g., by targeting only one epitope per antigen), whereas damage due to any activated complement could be mitigated by complement-inactivating agents (e.g., eculizumab); and damage due to ADCC might be addressed by suppression of natural killer (NK) cell activity. Such complications could pose barriers to regulatory approval, albeit perhaps less so for therapeutic versus prophylactic vaccines particularly where net benefit (e.g., gained by obviating ART for PLWH) would be strongly compelling. Nevertheless, either or both complement activation and ADCC still might contribute to net benefit if they actually decreased pathogen replication [e.g., possibly with antibodies to the scavenger receptor CD36, which have been shown to inhibit HIV-1 release from infected macrophages by clustering newly formed virions at their site of budding (10)].

Beyond HIV, other medically challenging infections likewise might be controlled through antibody targeting of host self epitopes. In a manner analogous to the blocking of HIV infection by antibodies that bind CD4, CCR4, or CCR5, infection due to other pathogens can be blocked by antibodies and antibody-like constructs that bind appropriate target epitopes on host cells; this is exemplified by the anti-infective activity of multivalent recombinant antibody fusion proteins that bind intercellular adhesion molecule 1 (ICAM-1), particularly against human rhinovirus (for which ICAM-1 serves as the major host receptor) (11). Anti-infective immunity also may be mediated by antibodies that selectively target modified-self epitopes of infected host cells, such as band-3 neotopes of parasitized erythrocytes in falciparum malaria (12). Yet another strategy might be the use of anti-idiotypic antibodies against infection-enhancing antibodies, which is potentially applicable where antibody-dependent enhancement (ADE) of infection [e.g., with HIV, a wide variety of other viruses and even cellular pathogens including bacteria and protozoa (13)] contributes to pathogenesis, although caution would be warranted to avoid adverse iatrogenic effects (e.g., autoimmune damage mediated by antibodies produced against the anti-idiotypic antibodies, resulting from molecular mimicry of host self epitopes by the anti-idiotypic antibodies).

The scheme described herein thus shifts the focus of immunity-oriented approaches in health care, from immune destruction of specific targets back to the original object of vaccination, namely host protection against disease. This widens the scope of vaccines and immunotherapeutics, by placing due emphasis on immune targeting of host self epitopes as a potential means for host protection associated with negligible or justifiably limited host damage. More generally, immune targeting of epitopes may be broadly conceptualized in terms of high-level functional outcomes including both familiar consequences of conventional immunization regimens (e.g., for prophylaxis or therapy primarily based on targeted immune destruction of microbial pathogens and host-derived malignant cells) as well as less obvious and possibly even counterintuitive but nonetheless beneficial results (e.g., host resistance to infections that is at least partly based on non-destructive binding of antibodies to host self epitopes). Such a perspective provides the basis for an expanded paradigm of biomedically enhanced immune function, the essence of which is concisely expressed as the idea of epitopes for protective immunity targeting antigens of pathogen and/or host (EPITAPH).

## Conflict of Interest Statement

The author declares that the research was conducted in the absence of any commercial or financial relationships that could be construed as a potential conflict of interest.
